# Sero-epidemiological studies of pathogen burden: A scoping review

**DOI:** 10.12688/wellcomeopenres.26070.3

**Published:** 2026-07-11

**Authors:** Anne Suffel, Danny Turton, Kathryin E Mansfield, Alexander J Mentzer, Naomi E Allen, Aisha Babi, David Burgner, Tim Waterboer, Charlotte Warren-Gash

**Affiliations:** 1 Department of Non-Communicable Disease Epidemiology, London School of Hygiene & Tropical Medicine, London, England, UK; 2 School of Health and Care Sciences, University of Lincoln, Lincoln, England, UK; 3 Centre for Human Genetics, University of Oxford, Oxford, England, UK; 4 Chinese Academy of Medical Science (CAMS) Oxford Institute (COI), University of Oxford, Oxford, England, UK; 5 Nuffield Department of Population Health, University of Oxford, Oxford, England, UK; 6 UK Biobank, Stockport, England, UK; 7 German Cancer Research Center, Heidelberg, Baden-Württemberg, Germany; 8 Faculty of Biosciences, Universitat Heidelberg, Heidelberg, Baden-Württemberg, Germany; 9 Royal Children’s Hospital, Murdoch Children's Research Institute, Parkville, Victoria, Australia

**Keywords:** pathogen burden; serology, infections

## Abstract

**Background:**

Methodological advances in serological testing have made it feasible to quantify antibodies to multiple pathogens simultaneously. This has been used to study the impact of pathogen burden, i.e., cumulative lifetime exposure to persistent pathogens, on health and mortality outcomes. However, definitions for pathogen burden using serological data vary considerably. It is unclear how to best combine serological data for multiple pathogens for exposure/outcome definitions in epidemiological studies.

**Methods:**

Following PRISMA-ScR guidelines, we conducted a scoping review of all relevant publications from MEDLINE, Embase, and Scopus until May 1 ^st^, 2025, with no language restrictions. We identified epidemiological studies defining pathogen burden, and which included at least three different pathogens.

**Results:**

We included a total of 74 studies from 19 countries (published 2000–2025). Serology data from 50 different pathogens were analysed across all studies, with pathogen burden measured by combining a range of 3–17 pathogens across studies into a single metric. Approaches included counts of seropositive result, quantiles of antibody titres, and more complex statistical analyses to group pathogens. No study gave a clinical/scientific rationale for the approaches chosen.

**Conclusions:**

Various analytical approaches have been used to define pathogen burden but there was limited justification for the chosen methodological approaches. More integrated approaches are needed for combining scientific and clinical knowledge of the interaction across different pathogens and epidemiological evidence into a meaningful measure of pathogen burden.

## Plain Language summary

Blood tests can show which infections a person has had in the past. These tests can check for many infections at the same time. However, there is no clear agreement on how to combine these results into one overall measure of a person’s past exposure to infections (pathogen burden).

We reviewed published studies to see how researchers have done this. We found 74 population health studies published between 2000 and 2025 that combined blood test results for at least three different infections. Across these studies, researchers looked at more than 50 infections and combined between 3 and 17 infections into a single score. Most studies simply counted how many infections a person had, while others used more complicated methods.

None of the studies clearly explained why they chose their method. Better ways are needed to measure exposure to multiple infections. These methods should be based on what we know about how infections affect the body and health, so the results are easier to understand and more useful.

## Introduction

Serological data are a useful tool for assessing an individual’s past exposure and immunity to pathogens. Various methods have been developed to examine serum or plasma for the presence of pathogen-specific antibodies. Laboratory techniques include functional assays (e.g., plaque or pseudovirus reduction neutralization assays [PRNT/pVNT]), and binding assays (including enzyme-linked immunosorbent assays [ELISA], fluorescent bead-based assays, or fluorescent micro-array-based assays) ^1^ or Phage Immunoprecipitation sequencing (PhIP-seq) technology. ^2^

Serology data provides a variety of advantages for epidemiological research. They are an objective measure that can detect asymptomatic or subclinical infections, and past infection ^3^ that does not rely on self-recall. In combination with other data sources, such as electronic health records and questionnaires, serology data can be used to investigate more complex and longitudinal interactions between various pathogens and chronic conditions. ^4, 5^

With the development of multiplex assays, it is possible to study previous exposure to multiple pathogens simultaneously from small blood samples, including archived dried blood spots. It uses beads coated with specific antibodies of binding sites for the pathogen which can then be read though flow cytometry using the fluorescent signature of the beads. ^6^ An increasing number of studies have investigated links between infections and various non-communicable diseases. ^7– 9^ Well-described associations include: the causal role of certain strains of human papillomaviruses and cervical and oropharyngeal cancer ^10^; *Helicobacter pylori* and gastric cancer ^11^; and Epstein Barr virus (EBV) and Burkitt’s lymphoma ^12^: EBV and multiple sclerosis ^13, 14^ and hepatitis viruses, and liver cirrhosis and hepatocellular carcinoma. ^15^

Often, more than one pathogen is linked to a chronic condition. For example, both *Chlamydophilia pneumoniae*, cytomegalovirus (CMV) and other herpes viruses, as well as several oral infections, have putatively been associated with atherosclerosis. ^9, 16^ The reactivation of the varicella zoster virus ^17^ and previous herpes simplex virus (HSV) 1 and 2, have both been associated with increased dementia risk. ^18^

A recent study found evidence for an association between multiple pathogens with non-communicable diseases, including replication of an association between cytomegalovirus and ulcerative colitis. ^8^ However, more research is needed on how multiple pathogens interact with each other to influence the pathogenesis of other health conditions. Understanding pathogen-disease interactions could help to improve prevention through targeted vaccination or treatment of infections. For example, infections in childhood have already shown to be associated with an increased risk of cardiovascular disease and cardiovascular risk factors in adults, offering opportunities for earlier prevention. ^19^ An optimal definition of pathogen burden could improve studies investigating the links between infection history and non-communicable disease outcomes. Furthermore, multiplex techniques could be used to detect viral epitopes that might not be required for defining a seropositive exposure status but are potentially associated with disease progression (“serolomics”). ^20^

We aimed to summarise how definitions of the burden of serologically defined infections have been conceptualised and operationalised in seroepidemiological studies to inform future research using measures of pathogen burden.

## Methods

We conducted our scoping review following guidelines outlined by the Preferred Reporting Items for Systematic Reviews and Meta-Analyses for Scoping Reviews (PRISMA-ScR ^21^). We sought to describe all methods used in epidemiological research to combine measurements of at least three different pathogens into a concept of pathogen burden (defined as a measure of cumulative exposure to at least three serologically defined pathogens).

### Review question

How are multiple serological measures of chronic or latent infections combined into a single concept, such as pathogen burden or infectious burden, for epidemiological research questions?

### Inclusion criteria

We included all peer-reviewed studies with an epidemiological research question in a human population that defined a composite measure of antibodies to at least three different pathogens. Previous infection with a pathogen had to be ascertained through serological data generated using any assay and at least three pathogens had to be combined into a single outcome or exposure. We chose studies with at least three pathogens to capture studies with more complex exposure/outcome definitions of multiple infections. There was no restriction on type of infection, country, year, or language of publication.

### Exclusion criteria

We excluded reviews, comments, opinion pieces, conference abstracts, study protocols, errata, grey literature, and retracted studies. Further, we excluded studies with less than three pathogens or if the prevalence or incidence was reported separately for each pathogen, rather than combined into a composite measure of pathogen burden. Studies with clinical, or other, definitions of infection but no serological data were excluded.

### Search strategy and study selection

For this scoping review, a literature search was conducted in MEDLINE, Embase, and Scopus from database inception to the 1 ^st^ of May 2025. The search strategy combined the concept of pathogen/infection burden with various kinds of infections and limited the study results to a human population. The specific infections in the search strategy focused on common persistent pathogens measured in studies investigating links between infections and non-communicable diseases (e.g., cardiovascular disease, cancer or dementia). The full search strategy can be found in the Supplement (S1 ^22^) refer to extended data. Titles were subsequently imported into Rayyan (web-based tool to screen and manage references during systematic reviews) for removal of duplicates, further screening and review. Two authors independently screened article titles and abstracts for inclusion based on the above criteria (AS and DT). All disagreements were resolved between the two authors (third author available if necessary). The full text of articles was subsequently reviewed for inclusion by two independent reviewers (AS and DT). Any discrepancies were discussed between the two authors for resolution.

### Data extraction and synthesis

The data from included articles were extracted by AS. Data from a 10% sample of the included studies were extracted by a second author (CWG) to ensure quality of the data extraction process. We extracted general study information, operationalisation of pathogen burden, pathogens included, serology methods used to assess past infection, and rationale for the choice of pathogen burden measure (Supplementary Text S1 ^22^ for more detail refer to extended data). If the location of the study was missing, we supplemented the location with the country of the institution the majority of authors were affiliated with. Extracted data were summarised narratively, and in tables and figures.

We did not conduct a risk of bias assessment of the studies as this review focused on the different conceptualisations of pathogen burden rather than the epidemiological questions addressed in the included studies.

### Role of the funding source

The funder had no role in study design, data collection, analysis, interpretation of the data, nor in writing the report and the decision to submit the paper for publication.

## Results

**Figure 1.**
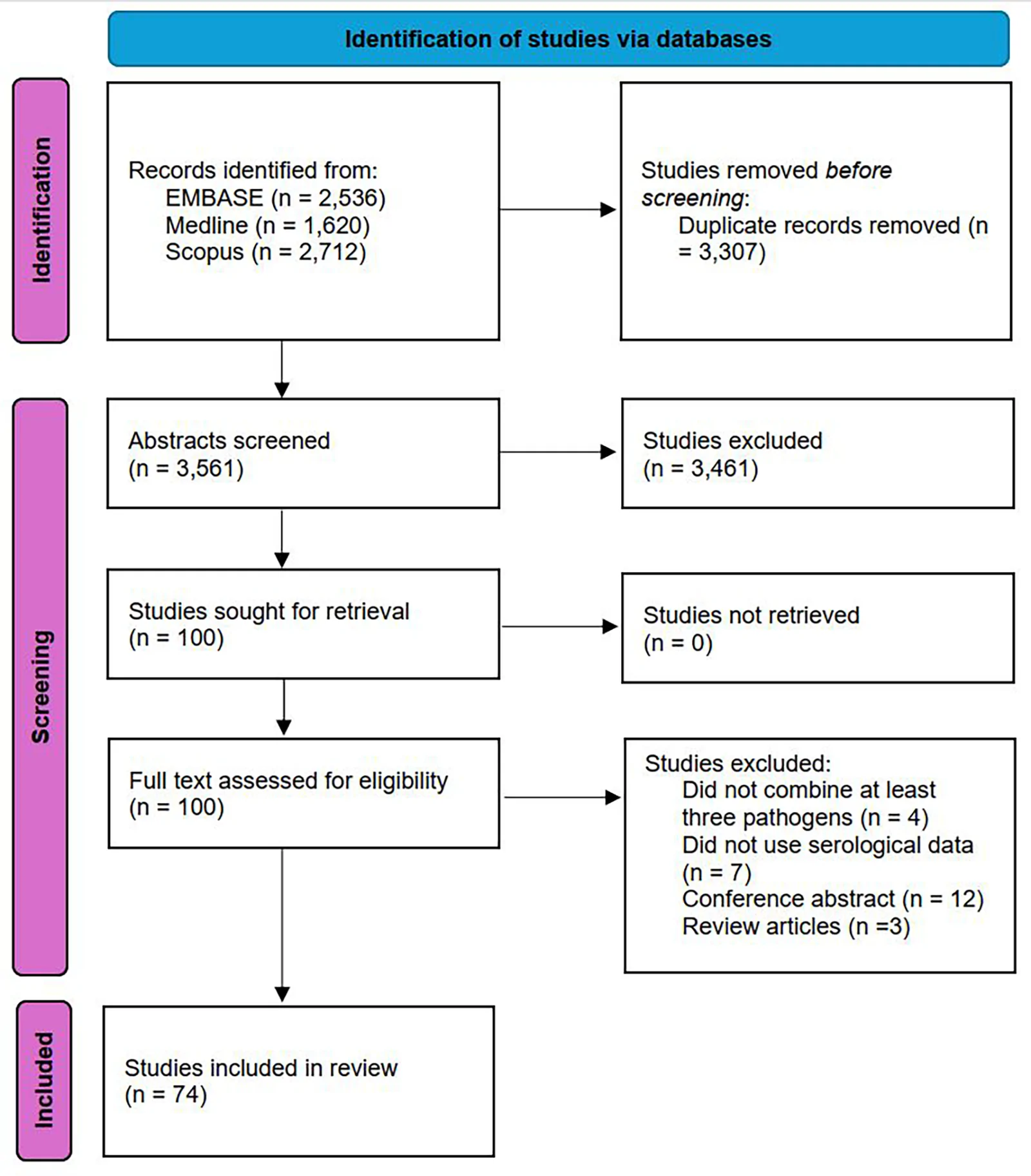
Number of articles retrieved and excluded at different stages of the review.

We retrieved 6,868 articles across all three databases ( Figure 1). Following removal of duplicates, 3,561 articles were included in abstract screening, 100 in full text screening, with 74 studies included in the final review (Table 1, refer to data repository ^22^).

### Summary of all studies

The 74 included studies were conducted in North America, Europe, or Asia. Most studies were conducted in the US (n studies = 23, 31.1% ^23– 45^), followed by Germany (n = 6, 8.1% ^46– 51^) and China (n = 6, 8.1% ^52– 57^). Six publications did not explicitly state the country where the study was conducted. ^58– 63^ Forty-four studies included cohorts of adults in a community setting (59.5% ^23, 26– 33, 35, 37– 45, 51, 63– 86^), 28 study populations were recruited adults from hospitals (37.8% ^24, 34, 46– 50, 52, 54– 60, 62, 87– 98^), and two studies included children (2.7% ^99, 100^).

The reporting of the pathogen burden measurement methods varied across different studies. Some studies (n = 34, 45.9%) reported details of the assays used including information on their sensitivity, specificity, and threshold values of measured antibody levels to define seropositivity. The most commonly used assay was Enzyme-Linked Immunosorbent Assay [ELISA] (n = 46, 62.2%, explicitly stated ^23, 24, 26, 27, 29– 31, 33– 40, 42, 44, 46– 50, 54– 57, 59, 62– 64, 68, 72, 76– 80, 82, 83, 85, 89– 92, 95, 101^). However, there were some studies that only referred to the commercial test kits (n = 3, 4.1% ^25, 75, 99^) or just stated immunoassays were used (n = 1, 1.4% ^66^). A full description of the assays, antibodies measured, population prevalence and cut-off values to define seropositivity can be found in the supplementary material.

**Figure 2.**
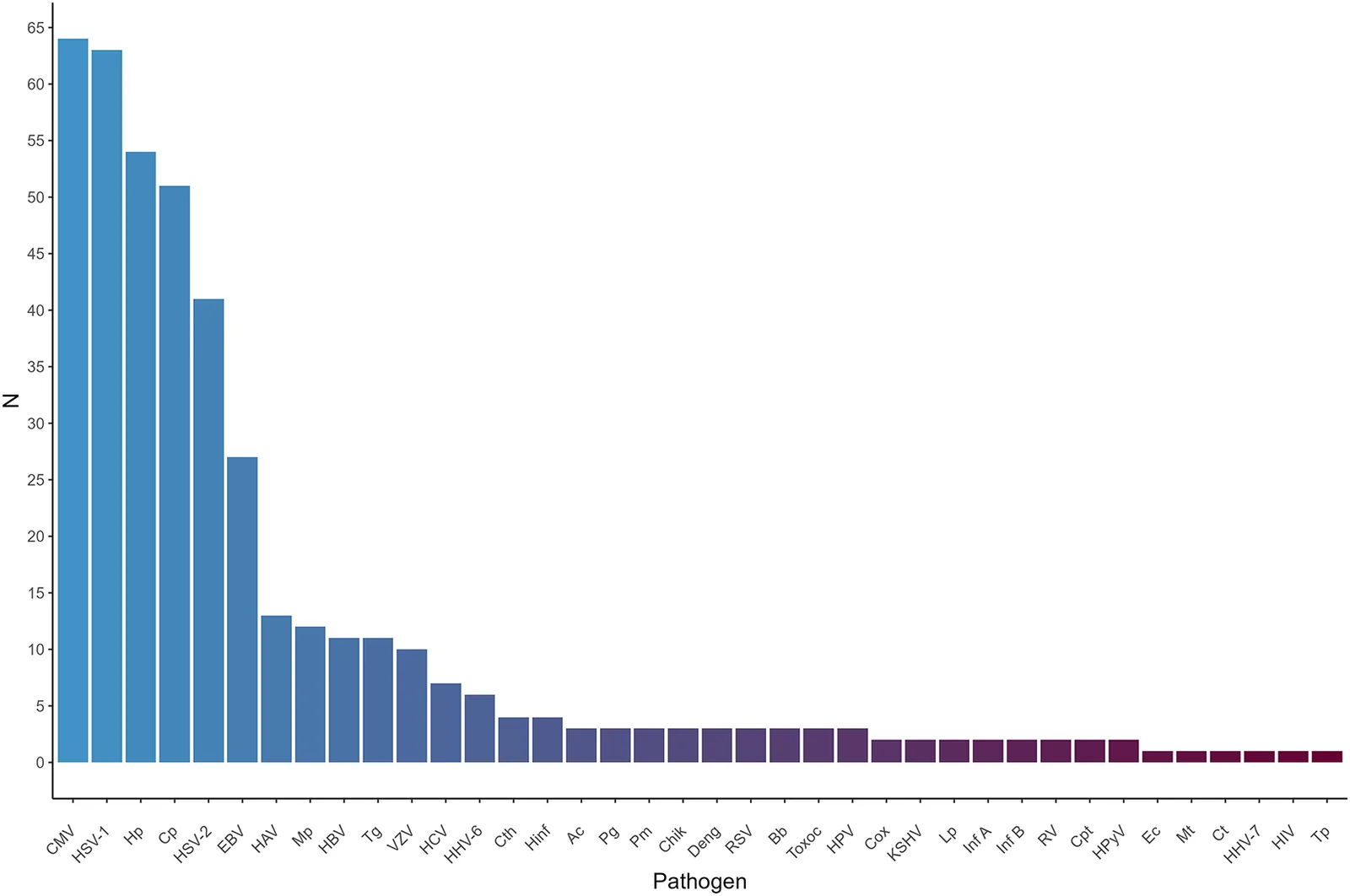
Frequency of different pathogens included in a pathogen burden measure across all studies. CMV: Cytomegalovirus. HSV-1: Herpes simplex virus 1. Hp: *Helicobacter pylori.* Cp: *Chlamydia pneumoniae.* HSV-2: Herpes simplex virus 2. EBV: Epstein-Barr virus. HAV: Hepatitis A virus. Mp: *Mycoplasma pneumoniae.* HBV: Hepatitis B virus. Tg: Toxoplasma gondii. VZV: Varicella zoster virus. HHV-6: Human herpesvirus 6 (A, B). HCV: Hepatitis C virus. Hinf: *Haemophilus influenzae.* Ac: *Aggregatibacter actinomycetemcomitans.* Pg: *Porphyromonas gingivalis.* Inf A: Influenza A virus. Inf B: Influenza B virus. Pm: Plasmodium malariae. HPV: Human papillomavirus (6, 11, 16, 18). Bb: *Borrelia burgdorferi.* Cox: Coxsackievirus B3. Ch t: *Chlamydia trachomatis.* RV: Rubella virus. Clt: *Clostridium tetani.* Chik: Chikungunya virus. Deng: Dengue virus. RSV: Respiratory syncytial virus. Toxoc: Toxocara canis and Toxocara cati. Ec: *Escherichia coli.* Lp: *Legionella pneumophila.* Mt: *Mycobacterium tuberculosis.* HPyV: Human polyomaviruses (6, 7, 9, 10). HHV-7: Human herpesvirus 7. KSHV: Kaposi’s Sarcoma-Associated Herpesvirus. HIV: human immunodeficiency virus. Syphilis: *Treponema pallidum.*

**Figure 3.**
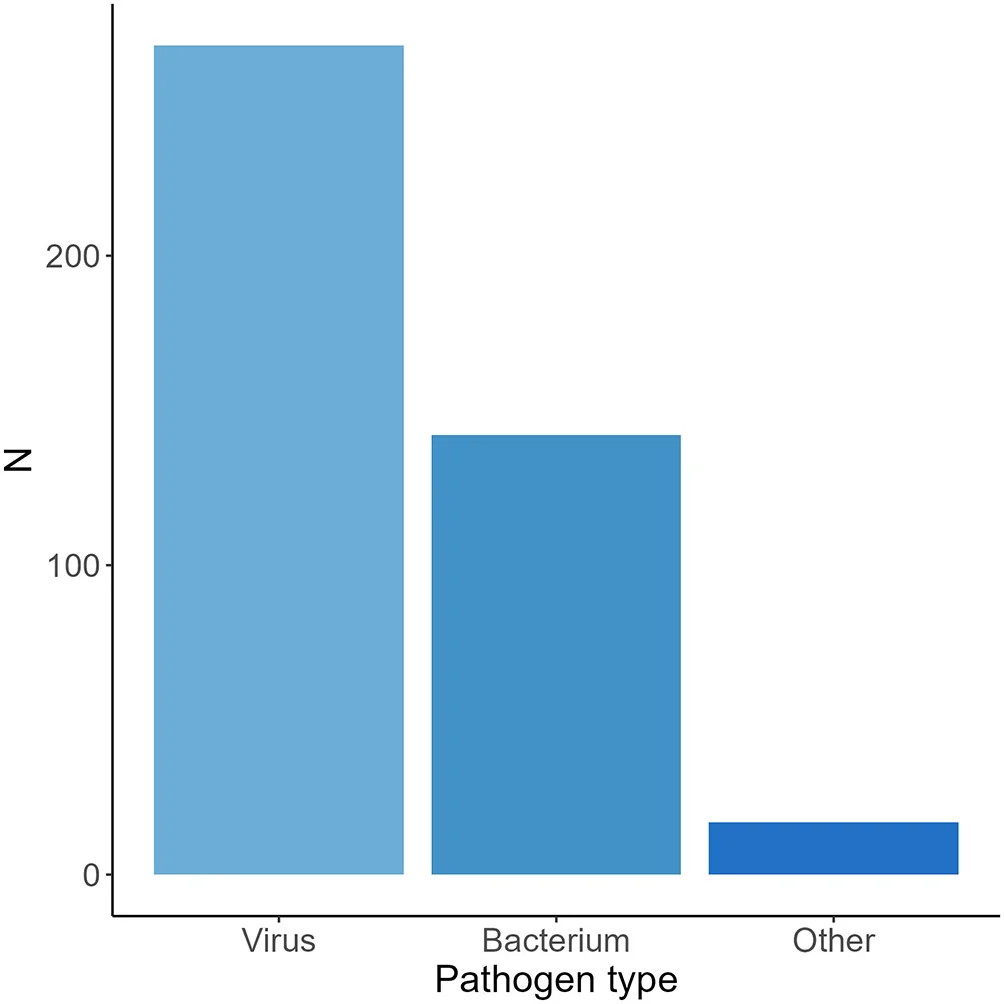
Types of pathogens included in pathogen burden concept across all included studies. The Other category includes: protozoae, helminths, and an alveolate.

**Figure 4.**
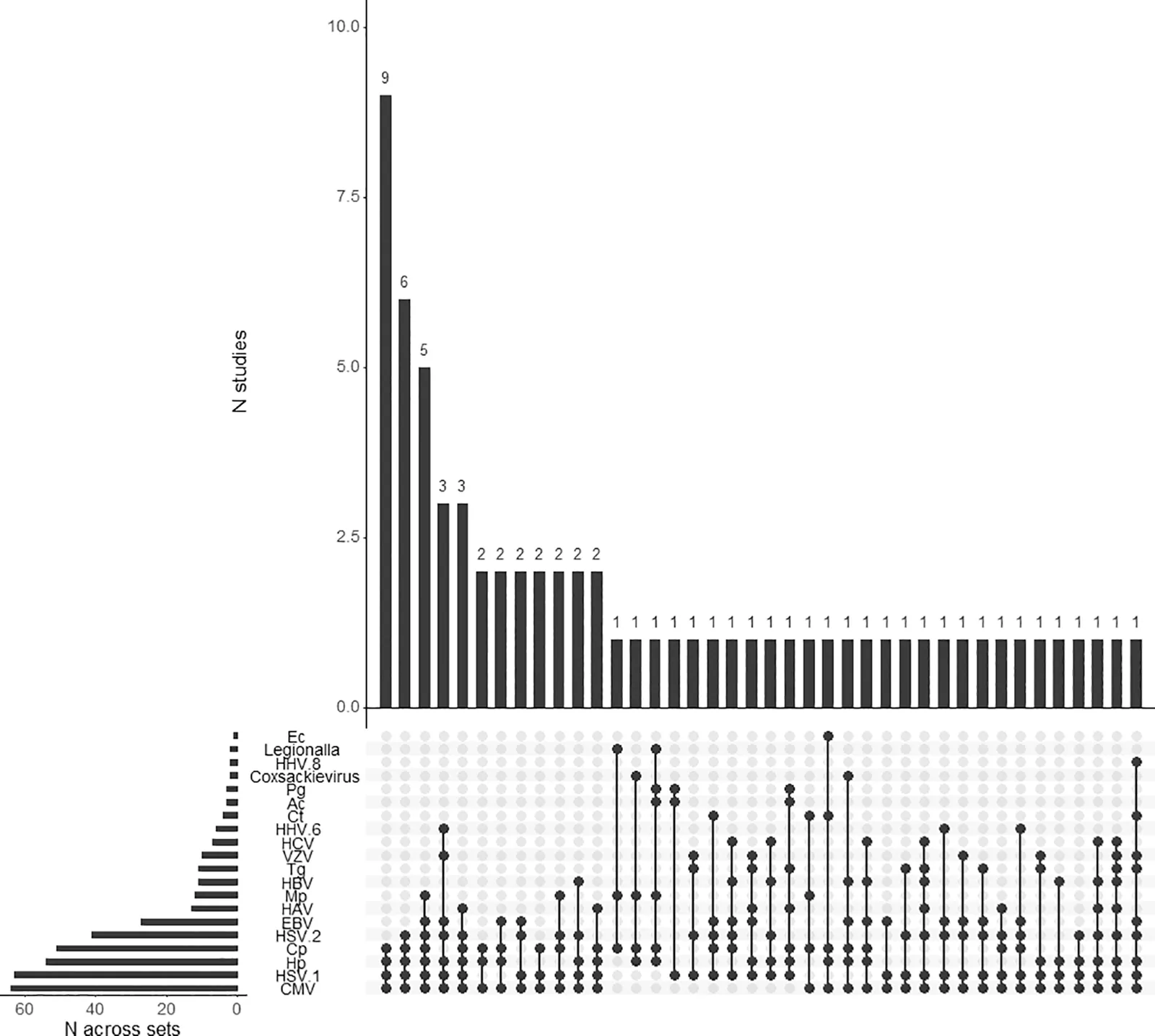
Frequency of combinations for the 20 most common pathogens included into pathogen burden across studies. N combinations states how many studies combined the pathogens under each bar, whereas N across sets indicates the frequency of each single pathogen across different studies. CMV: Cytomegalovirus. HSV-1: Herpes simplex virus 1. Hp: *Helicobacter pylori.* Cp: *Chlamydia pneumoniae.* HSV-2: Herpes simplex virus 2. EBV: Epstein-Barr virus. HAV: Hepatitis A virus. Mp: *Mycoplasma pneumoniae.* HBV: Hepatitis B virus. Tg: Toxoplasma gondii. VZV: Varicella zoster virus. HHV-6: Human herpesvirus 6 (A, B). HCV: Hepatitis C virus. Ct: *Chlamydia trachomatis.* H inf: *Haemophilus influenzae.* RSV: Respiratory syncytial virus. Ac: *Aggregatibacter actinomycetemcomitans.* Pg: *Porphyromonas gingivalis.* Inf A: Influenza A virus. Inf B: Influenza B virus.

Fifty pathogens were included across all studies, with the most studied pathogens being CMV (n = 64, 86.5% ^23– 31, 33– 44, 46, 48, 52, 54, 56– 60, 62– 65, 67– 70, 72, 73, 75– 84, 88– 92, 95, 97, 98, 100, 102, 103^), herpes simplex virus 1 (HSV-1, n = 63, 85.1% ^23– 31, 33– 46, 48, 54, 56, 57, 59, 62– 66, 68, 69, 72– 74, 76– 85, 87, 89, 90, 92, 95, 97– 99, 100, 102– 104^) and *H. pylori* (Hp, n = 54, 73.0% ^24, 26– 31, 33– 38, 40, 42– 44, 46, 48, 51– 54, 56, 58– 60, 62– 68, 70, 72, 73, 75– 77, 79, 82, 83, 88– 90, 92, 95, 97– 99, 102– 104^) ( Figure 2). The majority of pathogens were viruses (n = 268, 62.8% of measured pathogens), followed by bacteria (n = 142, 33.3%); only 17 (4.0%) measurements included other micro-organisms such as alveolates, protozoans, or helminths ( Figure 3). The number of pathogens combined into a putative ‘pathogen burden’ ranged from 3 to 17 across the studies. The pathogens most frequently combined into a single burden measure were CMV, HSV-1, Hp, and *Chlamydophila pneumoniae* (Cp); this combination measure was analysed in nine different studies ( Figure 4). Six studies of these nine studies using a combined CMV/HSV-1/Hp/Cp measure also included serological data on herpes simplex virus 2 (HSV-2).

The prevalence of each type of pathogen varied widely across different studies. For example, HSV-2 prevalence ranged from 1.5% in a paediatric study population ^100^ (Greece) to 74.5% in a population of individuals receiving haemodialysis ^98^ (country not stated).

Most studies (n = 71, 95.9%) included pathogen burden as an exposure, with the most studied outcomes being cognitive function, indicators of cardiovascular health, and inflammatory markers. Only three studies investigated pathogen burden as an outcome, investigating differences in socioeconomic and ethnic disparities in relation to prevalence of infections. ^28, 41, 105^

### Definitions of pathogen burden

Pathogen burden was assessed across different studies using various methods including using pathogen counts, combinations of seropositive status across specific pathogens, and antibody levels.

### Pathogen count

The majority of papers (n = 54, 73.0%, ^23– 26, 33– 35, 38, 42, 43, 46, 48– 50, 52– 56, 58– 60, 62– 64, 66– 68, 74, 78, 80– 84, 87– 91, 95– 98, 100, 106^) counted the number of pathogens each individual tested positive for and used the summed score as an exposure variable (53/54 studies). Depending on the prevalence of different pathogens, the baseline reference category for statistical analyses varied across studies, with the reference category usually comprised of scores of 0–2 or 0–3 positive counts (e.g., ^102, 107^). Some studies classified pathogen burden into low, medium or high exposure based on the absolute score (e.g., ^34, 66^). One study dichotomised pathogen score and defined the cut-off value based on its ability to predict the outcome using a receiver operator characteristic curve (ROC) curve analysis. ^57^ Two studies used the proportion of pathogens each individual tested positive as a proportion of all pathogens assayed. ^41, 65^ One study created a summary pathogen load coefficient that divided the sum of positive test results (pathogen load) by 1.6, which was the average summary pathogen load of the healthy comparator group. ^87, 93^

Seven studies (n = 7, 9.5%, ^35, 55, 56, 65, 80, 99, 100^) differentiated by the types of pathogens and created separate scores such as positive count for herpesviruses and polyomaviruses ^100^ or using a separate score for neurotropic viruses. ^65^

### Combinations of seropositive status

A single study with four different pathogens used all potential combinations of positive or negative serology tests across the four pathogens as their exposure variable. ^108^

### Use of antibody levels

Thirteen studies used antibody titres as an exposure variable (Table 1, refer to data repository ^22, 27, 29, 45, 51, 65, 72, 73, 75– 77, 85, 92, 107^). The majority (n = 11, 14.8%, ^27, 29, 45, 51, 65, 72, 73, 75– 77, 85, 92, 107^) of these studies divided antibody levels into thirds or fourths to define a categorical exposure variable for each pathogen separately. ^65, 109^ This individual-pathogen categorical exposure approach was used as an additional analysis for specific pathogens in addition to a composite measure. One study divided antibody levels for each pathogen into fourths and then assigned between 1–4 points for each pathogen depending on the category of each individual’s antibody titre level, using the total score as their exposure variable. ^85^ Another study used the average z-scored IgG antibody level across all five pathogens for all individuals who tested positive for at least one pathogen. ^31^ No study used a serological correlate of protection against a pathogen.

### Other pathogen burden definitions

Six studies (n = 6, 8.1%, ^30, 37, 39, 44, 86, 101^) used weighted regression coefficients as a pathogen score (i.e. all exposure and confounder variables were regressed on the outcome of interest, and a pathogen score calculated by summing the regression coefficients of each positive pathogen). ^30, 39, 101, 110^ One study used MIMIC models (multiple indicators, multiple causes), a type of structural equation modelling that captures the underlying statistical clusters of pathogens. ^28^ In this approach, the latent variable representing infection burden was modelled as influencing individual infection outcomes, and pathogen burden was expressed as a function of the observed predictors. Model fit was evaluated using the comparative fit index (CFI) and root mean square error of approximation (RMSEA). One study ^40^ used latent class analysis to identify common clusters of seropositivity status across different pathogens. They determined the number of classes by comparing different statistical models using a combination of likelihood-ratio value (G2), Akaike information criteria (AIC), Bayesian information criterion (BIC), sample size-adjusted BIC, log-likelihood and entropy. After the optimal number of classes was determined, they assigned a class to each individual based on their posterior probability of most likely class membership.

### Rationale behind pathogen scores

There was no scientific rationale described for the approach taken in any study. Some studies (n = 5, 6.8%, ^30, 37, 39, 44, 101^) referred to a review paper. ^16^ However, this paper only states that there is no consensus on defining pathogen burden and no clinical consensus about which pathogens should be included for pathogen burden score. A Russian study references a book chapter for their summary pathogen load definition that could not be verified by the authors of this review. ^93^

## Discussion

Our scoping review and previous related reviews in the literature, demonstrate that there is no consensus on how to define pathogen burden, nor for which pathogens to include in an index or score for pathogen burden. ^16^ We showed that there are major differences across studies in the choice of pathogens measured and how they are combined into a composite pathogen burden score. It remains unclear whether the wide range of pathogens included in studies reflected hypothesised associations with outcomes or highly prevalent pathogens in the study population, and whether it was also influenced by availability and cost of serological assays.

The approaches taken to define pathogen burden varied widely, ranging from simpler methods such as scores based on pathogen-specific seropositivity, to more complex methods such as latent class analysis and regression-based scores.

Using a summary score for different pathogens means that different pathogens are considered to contribute equally to the pathogenesis of a specific disease in the statistical model. However, this approach ignores potential synergistic effects between different pathogens as well unique pathological mechanisms. Only one study included in our review tried to address this issue by looking at the different combinations of positive serostatus across four pathogens. However, this approach may not always be feasible as the number of serostatus combinations increase exponentially with the number of pathogens. ^108^

Approaches such as the weighted pathogen index, which reported an association with the risk of stroke, ^111^ are difficult to interpret as the presented hazard ratios are in relation to the increase by one standard deviation of the index. The clinical significance of such a measure is unclear (e.g., what serostatus in an individual does this increased stroke risk correspond to?). Similar to using a simpler index, this approach also cannot differentiate potential synergistic and differing effects of different pathogens.

Latent class analysis provides a more nuanced approach to classifying different pathogens into exposure groups as it accounts for their relationships with the outcome, and other confounders. However, this method requires a sufficiently large sample size and may be limited when dealing with low prevalence infections. For example, in one study, HIV status did not contribute to class membership attribution owing to its low prevalence, ^40^ meaning that information from a potentially important infection was lost. This loss of information is particularly pertinent for HIV as it has been associated with higher risk of several non-communicable diseases including cardiovascular diseases, cancer, and chronic respiratory diseases. ^112, 113^ Furthermore, these methods do not acknowledge underlying biological hypotheses, e.g., tropism of the pathogen, and direct or indirect effects of the pathogens.

Using data on antibody titres may reflect the severity of the past infection, viral load, immunogenetics of the host and time since infection. However, this is highly dependent on the pathogen and antibody measured. Higher levels of haemagglutinin inhibition antibodies for influenza have been associated with febrile illness from influenza ^114^ and the antibody responses one year after SARS-CoV-2 infection was higher in individuals with more severe infections. ^115^ However, researchers found no association between the severity of mononucleosis and levels of specific EBV protein and neutralising antibodies after infection. ^116^ Higher antibody levels may also reflect a reactivation of a virus, for example, higher HSV-1 titres in individuals with more frequent cold sores. ^117^ Other pathogens such as *Neisseria gonorrhoeae* are difficult to prove due to great variability in antigens and often weak antibody response to infections. ^118^ It remains unclear how different levels of antibody responses are linked to the mechanisms involved in the pathogenesis of non-communicable diseases.

With the increasing availability of higher-volume and lower-cost testing assays for multiple pathogens, a challenge arises regarding how best to use these rich data to study combined effects of multiple pathogens on multiple disease outcomes. The choice of included pathogens in any measure of pathogen burden is highly dependent on the research question, study population of interest, and available laboratory technology for testing. Even within a multiplex assay, there may be different validities for different pathogens, which may be challenging for overall pathogen index validation. The study population of interest presents an additional challenge as children may not show a serological correlate of infection despite symptomatic disease. ^119^

Our scoping review included a wide range of different studies with no exclusion criteria related to country of study or publication language. Our search terms covered a wide range of pathogens often linked to non-communicable diseases, including commonly studied viruses and general terms for infections. However, our approach might have overlooked some rare or neglected pathogens. We also focused out search strategy on specified pathogens that have been commonly studies in relationship to non-communicable diseases and measures in large population-based cohorts such as the UK Biobank to inform future research using such resources. We did not assess risk of bias in the included studies as the main interest of this review was to assess the scope of methods used to conceptualise pathogen burden rather than to assess the quality of the epidemiological research.

Overall, a better integration of knowledge of the infection pathogenicity, transmission route, and epidemiology, in relation to underlying cellular and genetic mechanisms of the disease (outcome) of interest, are essential for better operationalisation of pathogen burden in epidemiological research. No universal indicator of pathogen burden will be feasible as long there is no agreed set of pathogens that should be tested for in epidemiological research. As a consequence, some findings of studies using their own definition of pathogen burden may not be reproducible unless the same definition and method of defining pathogen burden is used. Future studies should include a clear justification for the measure of pathogen burden used as well as a clear description of both serological assays and statistical methods (see key recommendations).

Key recommendations for reporting pathogen burden in epidemiological studies:

- Clearly defined research question and explanation of the role of pathogen burden (e.g., outcome, exposure, covariate)
- List of pathogens included
- Justification for choice of pathogens included (e.g., relevance to study population)
- Reporting of type of assays, and thresholds used to define sero-positivity
- Any other supplementary methods used to measure pathogens (e.g., clinical data, surveys)
- Statistical methods used to aggregate serological data
- Justifications for the methods used (e.g., choice of assays, thresholds, statistical approach)
- Careful interpretations of findings in the context of the selected pathogens and research question
- Comment on the generalisability of findings

## Ethics and consent

Ethical approval and consent were not required.

## Data Availability

Extended data and study protocol can be found in the supplementary material: https://doi.org/10.5281/zenodo.18669395. ^22^ Data are available under the terms of the MIT License.
