# Supplementary material for: Sero-epidemiological studies of pathogen burden: A scoping review
Source: Wellcome Open Res. 2026 Jul 11;11:192. Originally published 2026 Mar 26. [Version 3] doi: 10.12688/wellcomeopenres.26070.3 (PMC13554841; doi:10.12688/wellcomeopenres.26070.3)
Supplement: Peer review history

# Reviewer report: Reviewer response for version 3

**DOI:** 10.21956/wellcomeopenres.29880.r160643

**Published:** 2026-10-23

**Authors:** Gary Lin

**Affiliations:** 1 Johns Hopkins University Applied Physics Laboratory, Laurel, Maryland, USA

I have reviewed the response and the paper has sufficiently addressed my concerns.

Are the rationale for, and objectives of, the Systematic Review clearly stated?

Yes

Is the statistical analysis and its interpretation appropriate?

Yes

If this is a Living Systematic Review, is the ‘living’ method appropriate and is the search schedule clearly defined and justified? (‘Living Systematic Review’ or a variation of this term should be included in the title.)

Not applicable

Are sufficient details of the methods and analysis provided to allow replication by others?

Partly

Are the conclusions drawn adequately supported by the results presented in the review?

Yes

Reviewer Expertise:

Computational Epidemiology, Public Health, and Systems Modeling and Simulation

I confirm that I have read this submission and believe that I have an appropriate level of expertise to confirm that it is of an acceptable scientific standard.

# Reviewer report: Reviewer response for version 3

**DOI:** 10.21956/wellcomeopenres.29880.r161004

**Published:** 2026-07-22

**Authors:** Andrea Haselbeck

**Affiliations:** 1 The University of Edinburgh, Edinburgh, Scotland, UK

This scoping review, conducted according to PRISMA-ScR, characterises how epidemiological studies have combined serological data on three or more pathogens into a composite measure of "pathogen burden." Searching MEDLINE, Embase and Scopus to 1 May 2025 without language restriction, the authors screened 3,561 records and included 74 studies published between 2000 and 2025, spanning 19 countries and 50 pathogens.

 This is a useful and well-motivated review addressing a real and under-recognised methodological problem.

 My only reservation would be that this review seems to focus on non-communicable disease, as mentioned briefly in the search strategy "links between infections and NCDs. This should be acknowledge in title or in the background for clarity. A stratified presentation by pathogen (viral vs bacterial vs others) is done, but then not further followed through. I would be interested if definitions of pathogen burden are more aligned within one pathogen grouping as compared to just overall. Has it been considered to distinguish between pathogens for which seropositivity is a lifetime marker versus for which it vanishes? There is no mention of this in methods or the paragraph on use of antibody levels in the results.

Are the rationale for, and objectives of, the Systematic Review clearly stated?

Yes

Is the statistical analysis and its interpretation appropriate?

Yes

If this is a Living Systematic Review, is the ‘living’ method appropriate and is the search schedule clearly defined and justified? (‘Living Systematic Review’ or a variation of this term should be included in the title.)

Not applicable

Are sufficient details of the methods and analysis provided to allow replication by others?

Partly

Are the conclusions drawn adequately supported by the results presented in the review?

Yes

Reviewer Expertise:

Epipdemiological studies, disease surveillance

I confirm that I have read this submission and believe that I have an appropriate level of expertise to confirm that it is of an acceptable scientific standard.

# Response

**Published:** 2026-08-13

**Authors:** Anne Suffel

**Affiliations:** Department of Non-Communicable Disease Epidemiolog, London School of Hygiene and Tropical Medicine Faculty of Epidemiology and Population Health, London, England, UK

**Comment 1: ** This scoping review, conducted according to PRISMA-ScR, characterises how epidemiological studies have combined serological data on three or more pathogens into a composite measure of "pathogen burden." Searching MEDLINE, Embase and Scopus to 1 May 2025 without language restriction, the authors screened 3,561 records and included 74 studies published between 2000 and 2025, spanning 19 countries and 50 pathogens.

 This is a useful and well-motivated review addressing a real and under-recognised methodological problem.

 My only reservation would be that this review seems to focus on non-communicable disease, as mentioned briefly in the search strategy "links between infections and NCDs. This should be acknowledge in title or in the background for clarity.

**Author’s response:** We added a sentence about the focus on NCDs in Version 3 of the manuscript which states the following: “An optimal definition of pathogen burden could improve studies investigating the links between infection history and non-communicable disease outcomes.” We also stated this more explicitly in the aims of the study: “We aimed to summarise how definitions of the burden of serologically defined infections have been conceptualised and operationalised in seroepidemiological studies to inform future research using measures of pathogen burden in relation to non-communicable diseases.”

**Comment 2:** A stratified presentation by pathogen (viral vs bacterial vs others) is done, but then not further followed through. I would be interested if definitions of pathogen burden are more aligned within one pathogen grouping as compared to just overall.

**Author’s response:** We included an additional paragraph differentiating between the types of pathogens included in the definition of pathogen count. “ ***Pathogen burden by types of pathogens*** The majority of studies included a mix of bacteria and viruses in their definition of pathogen burden independent of their approach how to define pathogen burden. Five studies using pathogen counts included viruses only (Jafarzadeh et al., 2011; Jonker et al., 2017; Karachaliou et al., 2018; Miller et al., 2014; Steptoe et al., 2007) and one study using counts included bacteria only (Ngeh & Goodbourn, 2005).  Out of the studies using a mixture of viruses, bacteria and other pathogens, six used a pathogen count (Alcantara-Neves et al., 2012; Janson et al., 2007; Lobzin et al., 2005; Pearce et al., 2018; Rubicz et al., 2015; van der Ven et al., 2003), five used the titers of pathogens for their index (Beydoun et al., 2023; Green et al., 2024; Lu et al., 2021, 2022; Shi et al., 2024) and five studies used other statistical methods (Gale et al., 2016; Hedges et al., 2017; Sealy-Jefferson et al., 2013; Stebbins et al., 2019; Zeng et al., 2025).”

**Comment 3:** Has it been considered to distinguish between pathogens for which seropositivity is a lifetime marker versus for which it vanishes? There is no mention of this in methods or the paragraph on use of antibody levels in the results.

**Author’s response:** The primary focus of this review was to understand different methodological approaches taken to define pathogen burden in epidemiological research. We agree that it would be interesting to distinguish between pathogens with serological lifetime markers versus those with waning antibody levels. To differentiate between pathogens with lifetime antibody markers and those without is quite challenging. For some pathogens such as Helicobacter pylori this is highly dependent on the antigen itself, some pathogens with more local infections such as HPV tend to have lower antibody titres in general. However, we acknowledge that especially for acute respiratory and gastrointestinal pathogens, quickly waning antibody responses are often observed. Hence, we decided to add this to the result sections. “Three studies including pathogens with shorter-lived antibody responses such as RSV used antibody titres to define pathogen burden (Lu et al., 2021, 2022; Shi et al., 2024). In contrast, two studies used counts of seropositivity for influenza viruses (Niu et al., 2019; Rubicz et al., 2015).”

# Reviewer report: Reviewer response for version 3

**DOI:** 10.21956/wellcomeopenres.29880.r160644

**Published:** 2026-07-21

**Authors:** Timothy R Julian

**Affiliations:** 1 Eawag Swiss Federal Institute of Aquatic Science and Technology, Dübendorf, Zurich, Switzerland

The authors have included discussion points in response to the previous comments, addressing the priority points for improving clarity.

 One issue remains, which is the disconnect between the very specific focus on "persistent pathogens associated with non-communicable diseases" and the very general title and framing of the review as a "scoping review of Sero-epidemiological studies of pathogen burden".

 Although some language in the literature search as the author notes is general, there is also a substantial focus on the subset of persistent pathogens that are explicitly searched for.  This manifests, for example, in the substantial representation of CMV.  

 A more accurate review article would highlight the focus of the search and the results on persistent pathogens associated with noncommunicable diseases in the title, abstract, introduction, and discussion.

 This minor framing for clarify and specificity is the only outstanding concern in an otherwise well written and well documented assessment of pathogen burden.

Are the rationale for, and objectives of, the Systematic Review clearly stated?

Yes

Is the statistical analysis and its interpretation appropriate?

Yes

If this is a Living Systematic Review, is the ‘living’ method appropriate and is the search schedule clearly defined and justified? (‘Living Systematic Review’ or a variation of this term should be included in the title.)

Not applicable

Are sufficient details of the methods and analysis provided to allow replication by others?

Yes

Are the conclusions drawn adequately supported by the results presented in the review?

Partly

Reviewer Expertise:

environmental microbiology, pathogen surveillance

I confirm that I have read this submission and believe that I have an appropriate level of expertise to confirm that it is of an acceptable scientific standard.

# Reviewer report: Reviewer response for version 1

**DOI:** 10.21956/wellcomeopenres.28710.r152401

**Published:** 2026-06-06

**Authors:** Timothy R Julian

**Affiliations:** 1 Eawag Swiss Federal Institute of Aquatic Science and Technology, Dübendorf, Zurich, Switzerland

Overall:  The authors provide an interesting review of published literature that reports use of composite variables derived from aggregating multiple pathogen-specific serology results to inform "pathogen" or "infectious disease burden" indicator.  The study is clear, with well described methodology, and initiates an important conversation on how to aggregate serology data in studies investigating multiple pathogens.

 The authors should explicitly list the pathogens included in the search criteria, and motivate why they chose this subset of pathogens.  The list included appears narrow, but the overall manuscript reflects a more general set of conclusions.  This should be resolved by narrowing the manuscript narrative to the subset of pathogens chosen. See detailed comments.

 Also, it is not clear to me that serology data from multiple distinct pathogens should be aggregated without a clear mechanistic model or motivation for the development of a composite indicator.  Authors could clarify this in the discussion section: under which conditions should analyses be done on composite variables, as oppose to on individual pathogen data?

 Detailed Comments:

 Abstract background: Its unclear to me what pathogen burden is in the abstract, could it also be defined conceptually in the background?

 Abstract results:  Why 3-17?  Are these the number of pathogens tested?  This could be clarified here.  With pathogen burden measured by combining the analyzed pathogens (from 3-17 across studies) into a single metric.

 Conceptual comment: pathogen burden will be defined by the subset of pathogens investigated; within that context, no universal indicator is feasible until and unless every study follows the same set of pathogens tested.

 Data extraction: It may be helpful to have a second author review a subset of the extracted data to ensure consistency and accuracy.

 Introduction:

 I don't understand the sentence about serolomics.  Can authors expand on this concept?  If viral epitopes are detected, why is this not considered seropositive?

 Methods:

 I am missing an explicit definition of pathogen burden or infectious burden, and I am also missing the indicator values (what is measured) for pathogen or infecctious burden.  Can you provide definitions and indicators here, not only by you but also what is included in the publications assessed?  

 Summary of all studies: have you considered author affiliations for insight on the location of the studies, or contacting authors?  This would be helpful information to obtain.

 Of the forty-four adult studies and two child studies: did the rest not report or were they mixed age studies?

 Title / Content mismatch: There is a significant mismatch between the general title topic of "pathogen burden" and the vary narrow set of pathogens included in the search strategy (See Supplemental).  This manifests in a very skewed interpretation of relevant pathogens, as CMV appears in 86% of all included studies.  Missing are studies on, for example, respiratory or gastrointestinal diseases.  Authors should consider refining the title and indeed the overall framing to focus specifically on this subset of investigated pathogens (oncogenic and ulcerative pathogens?).  It is also not explicit why these specific pathogens were chosen and others were neglected.  In my opinion, not limiting search criteria to a set of pathogens would result in a more comprehensive analyses, though I understand this would be substantially more work.

 Discussion: "unique pathological mechanisms": also, using this approach will bias findings to the specific (often limited) set of pathogens chosen, as a consequence the findings may not be meaningfully reproducible unless others use the exact same set of pathogens and develop the composite score in the same way.  

 Using data on antibody titres: to what extent is this also biased by recency of infection?  How does this hidden component of time since infection shift study findings?

 "The choice of included pathogens" - I would argue that aggregation of serology data from multiple pathogens should be done in careful consideration of mechanisms, or otherwise should not be done at all.  That is, authors should analyse data per pathogen; if multiple pathogen burden is the research question, then the specific choice of pathogens included and the development of the composite variable should be grounded in theory of the pathogen interactions.

 "Our search terms covered a wide range… " see previous comment; I would argue this is a quite limited set of pathogens.  Missing are common endemic diseases.

Are the rationale for, and objectives of, the Systematic Review clearly stated?

Yes

Is the statistical analysis and its interpretation appropriate?

Yes

If this is a Living Systematic Review, is the ‘living’ method appropriate and is the search schedule clearly defined and justified? (‘Living Systematic Review’ or a variation of this term should be included in the title.)

Not applicable

Are sufficient details of the methods and analysis provided to allow replication by others?

Yes

Are the conclusions drawn adequately supported by the results presented in the review?

Partly

Reviewer Expertise:

infectious disease transmission, environmental engineering, environmental microbiology

I confirm that I have read this submission and believe that I have an appropriate level of expertise to confirm that it is of an acceptable scientific standard, however I have significant reservations, as outlined above.

# Response

**Published:** 2026-06-24

**Authors:** Anne Suffel

**Affiliations:** Department of Non-Communicable Disease Epidemiolog, London School of Hygiene and Tropical Medicine Faculty of Epidemiology and Population Health, London, England, UK

**Comment 1: Abstract background: Its unclear to me what pathogen burden is in the abstract, could it also be defined conceptually in the background?**

**Author's response:** We added a definition of pathogen burden in the background of the abstract. “Background Methodological advances in serological testing have made it feasible to quantify antibodies to multiple pathogens simultaneously. This has been used to study the impact of pathogen burden, i.e. the cumulative lifetime exposure to persistent pathogens, on health and mortality outcomes. However, definitions for pathogen burden using serological data vary considerably. It is unclear how to best combine serological data for multiple pathogens for exposure/outcome definitions in epidemiological studies.”

**Comment 2: Abstract results:  Why 3-17?  Are these the number of pathogens tested?  This could be clarified here.  With pathogen burden measured by combining the analyzed pathogens (from 3-17 across studies) into a single metric.**

**Author's response: **We clarified this in the abstract: “Serology data from 50 different pathogens were analysed across all studies, with pathogen burden measured by combining a range of 3–17 pathogens across studies into a single metric.”

**Comment 3: Conceptual comment: pathogen burden will be defined by the subset of pathogens investigated; within that context, no universal indicator is feasible until and unless every study follows the same set of pathogens tested.**

**Author's response:** We agree with the reviewer and added this consideration as a sentence into the discussion of the paper. “Overall, a better integration of knowledge of the infection pathogenicity, transmission route, and epidemiology, in relation to underlying cellular and genetic mechanisms of the disease (outcome) of interest, are essential for better operationalisation of pathogen burden in epidemiological research. No universal indicator of pathogen burden will be feasible as long there is no agreed set of pathogens that should be tested for in epidemiological research. As a consequence, some findings of studies using their own definition of pathogen burden may not be reproducible unless the same definition and method of defining pathogen burden is used. Future studies should include a clear justification for the measure of pathogen burden used as well as a clear description of both serological assays and statistical methods (see key recommendations).”

**Comment 4: Data extraction: It may be helpful to have a second author review a subset of the extracted data to ensure consistency and accuracy.**

**Author's response: **We had a second author reviewing a 10% subset of the extracted data. We added this to the description of the methods as well. “The data from included articles were extracted by AS. Data from a 10% sample of the included studies were extracted by a second author (CWG) to ensure quality of the data extraction process. We extracted general study information, operationalisation of pathogen burden, pathogens included, serology methods used to assess past infection, and rationale for the choice of pathogen burden measure ( **Supplementary Text S1** ^22^ for more detail refer to extended data). Extracted data were summarised narratively, and in tables and figures.”

****

**Comment 5: Methods:**

**I am missing an explicit definition of pathogen burden or infectious burden, and I am also missing the indicator values (what is measured) for pathogen or infectious burden.  Can you provide definitions and indicators here, not only by you but also what is included in the publications assessed?**  

**Author's response:** We have now added text to clarify the definition of pathogen burden in the methods and the inclusion criteria. In addition, we present the individual assays included in each pathogen burden measure by pathogen for each study, along with cut off values ad prevalence where available in supplementary material ( https://doi.org/10.5281/zenodo.18669395: Supplementary_table_antibody_assays.xlsx) “We sought to describe all methods used in epidemiological research to combine measurements of at least three different pathogens into an instrument of pathogen burden (defined as a measure of cumulative exposure to at least three serologically defined pathogens)” “We included all peer-reviewed studies with an epidemiological research question in a human population that defined a composite measure of antibodies to at least three different pathogens. Previous infection with a pathogen had to be ascertained through serological data generated using any assay and at least three pathogens had to be combined into a single outcome or exposure.

**Comment 6: Summary of all studies: have you considered author affiliations for insight on the location of the studies, or contacting authors?  This would be helpful information to obtain.**

**Author's response:** 6 studies had missing information on the study’s location. We have now used the author’s affiliations to supplement this information in Table 1 and also edited the manuscript accordingly. As all authors of these papers were affiliated to institutions from the same country, this approach seemed appropriate.   “The data from included articles were extracted by AS. Data from a 10% sample of the included studies were extracted by a second author (CWG) to ensure quality of the data extraction process. We extracted general study information, operationalisation of pathogen burden, pathogens included, serology methods used to assess past infection, and rationale for the choice of pathogen burden measure ( **Supplementary Text S1** ^22^ for more detail refer to extended data). If the location of the study was missing, we supplemented the location with the country of the institution the majority of authors were affiliated with.  Extracted data were summarised narratively, and in tables and figures.”  

**Comment 7: Of the forty-four adult studies and two child studies: did the rest not report or were they mixed age studies?**

**Author's response:** We clarified the description of the study population, now explicitly stating the study populations in hospitals were also adults. We abstained from differentiating more by age in the adults population, as the age spans in the adult populations varied widely and we did not want to impose a definition of “senior” population. **“**Forty-four studies included cohorts of adults in a community setting (59.5%, ^23^^, ^^26^^– ^^33^^, ^^35^^, ^^37^^– ^^45^^, ^^51^^, ^^63^^– ^^86^ ), 28 study populations recruited adults from  hospitals (37.8% ^24^^, ^^34^^, ^^46^^– ^^50^^, ^^52^^, ^^54^^– ^^60^^, ^^62^^, ^^87^^– ^^98^ ), and two studies included children (2.7% ^99^^, ^^100^ ).”

**Comment 8: Title / Content mismatch: There is a significant mismatch between the general title topic of "pathogen burden" and the vary narrow set of pathogens included in the search strategy (See Supplemental).  This manifests in a very skewed interpretation of relevant pathogens, as CMV appears in 86% of all included studies.  Missing are studies on, for example, respiratory or gastrointestinal diseases.  Authors should consider refining the title and indeed the overall framing to focus specifically on this subset of investigated pathogens (oncogenic and ulcerative pathogens?).  It is also not explicit why these specific pathogens were chosen and others were neglected.  In my opinion, not limiting search criteria to a set of pathogens would result in a more comprehensive analyses, though I understand this would be substantially more work.**

**Author's response: **This scoping review aimed to inform studies investigating the link between multiple persistent pathogens defined serologically and non-communicable diseases, with a focus on pathogens that have been tested in large population-based cohorts such as UK Biobank. Acute respiratory and gastrointestinal infections are rarely included in multiplex serology panels in these cohorts. Nevertheless, we also searched for more general terms such as ‘pathogen or infection burden’, which would  capture studies including a wider variety of pathogens. We added some explanation for the pathogens explicitly included in the search terms. “For this scoping review, a literature search was conducted in MEDLINE, Embase, and Scopus from database inception to the 1 ^st^ of May 2025. The search strategy combined the concept of pathogen/infection burden with various kinds of infections and limited the study results to a human population. The specific infections in the search strategy focused on common persistent pathogens measured in studies investigating links between infections and non-communicable diseases (e.g., cardiovascular disease, cancer or dementia). The full search strategy can be found in the Supplement ( **S1** ^22^ ) refer to extended data.” We also added this as a limitation to our discussion section: “ We also focused our search strategy on specified pathogens that have been commonly studied in relationship to non-communicable diseases and measured in large population-based cohorts such as UK Biobank to inform future research using such resources“

**Comment 9: "unique pathological mechanisms": also, using this approach will bias findings to the specific (often limited) set of pathogens chosen, as a consequence the findings may not be meaningfully reproducible unless others use the exact same set of pathogens and develop the composite score in the same way.**  

**Author's response: **We agree with the reviewer and added this to the limitations of our study in the discussion section: “Overall, a better integration of knowledge of the infection pathogenicity, transmission route, and epidemiology, in relation to underlying cellular and genetic mechanisms of the disease (outcome) of interest, are essential for better operationalisation of pathogen burden in epidemiological research. No universal indicator of pathogen will be feasible as long there is no agreed set of pathogens that should be tested for in epidemiological research.  As a consequence, some findings of studies using their own definition of pathogen burden may not be reproducible unless the same definition and method of defining pathogen burden is used.”

**Comment 10: Using data on antibody titres: to what extent is this also biased by recency of infection?  How does this hidden component of time since infection shift study findings?**

**Author's response: **We discuss these limitations in the discussion but improved the clarity of this point: **“**Using data on antibody titres may reflect the severity of the past infection, viral load, immunogenetics of the host and time since infection. However, this is highly dependent on the pathogen and antibody measured. Higher levels of haemagglutinin inhibition antibodies for influenza have been associated with febrile illness from influenza ^114^ and the antibody responses one year after SARS-CoV-2 infection was higher in individuals with more severe infections. ^115^ However, researchers found no association between the severity of mononucleosis and levels of specific EBV protein and neutralising antibodies after infection. ^116^ Higher antibody levels may also reflect a reactivation of a virus, for example, higher HSV-1 titres in individuals with more frequent cold sores. ^117^ Other pathogens such as *Neisseria gonorrhoeae* are difficult to prove due to great variability in antigens and often weak antibody response to infections. ^118^ It remains unclear how different levels of antibody responses are linked to the mechanisms involved in the pathogenesis of non-communicable diseases.”

**Comment 11: "The choice of included pathogens" - I would argue that aggregation of serology data from multiple pathogens should be done in careful consideration of mechanisms, or otherwise should not be done at all.  That is, authors should analyse data per pathogen; if multiple pathogen burden is the research question, then the specific choice of pathogens included and the development of the composite variable should be grounded in theory of the pathogen interactions.**

**Author's response:** We share the reviewer’s concern around missing theories around pathogen interactions. However, we also believe that creating a composite measure of multiple pathogens can help to develop hypotheses for how pathogens interact with each other and investigate potential mechanisms. For some of the included pathogens in our study, the detailed mechanisms of interaction with the human host, other pathogens or their tropism are not fully understood. Whereas many studies have investigated relationships between single pathogens and health outcomes, we believe that examining health outcomes in relation to multiple pathogens can add knowledge about potential interactions between multiple pathogens.   We added another key recommendation to reflect this:   “Key recommendations for reporting pathogen burden in epidemiological studies:

-  Clearly defined research question and explanation of the  role of pathogen burden (e.g., outcome, exposure, covariate)
- List of pathogens included
- Justification for choice of pathogens included (e.g., relevance to study population)
- Reporting of type of assays, and thresholds used to define sero-positivity
- Any other supplementary methods used to measure pathogens (e.g., clinical data, surveys)
- Statistical methods used to aggregate serological data
- Justifications for the methods used (e.g., choice of assays, thresholds, statistical approach)
- Careful interpretations of findings in the context of the selected pathogens and research question
- Comment on the generalisability of findings

”

**Comment 12: "Our search terms covered a wide range… " see previous comment; I would argue this is a quite limited set of pathogens.  Missing are common endemic diseases.**

**Author's response:** We edited our strengths and limitations section in the discussion to better reflect what kind of pathogens we included in our search criteria. “Our scoping review included a wide range of different studies with no exclusion criteria related to country of study or publication language. Our search terms covered a wide range of pathogens often linked to non-communicable diseases, including commonly studied viruses and general terms for infections. However, our approach might have overlooked some rare or neglected pathogens. We also focused our search strategy on specified pathogens that have been commonly studied in relationship to non-communicable diseases and measured in large population-based cohorts such as UK Biobank to inform future research using such resources”

# Reviewer report: Reviewer response for version 1

**DOI:** 10.21956/wellcomeopenres.28710.r151934

**Published:** 2026-05-05

**Authors:** Gary Lin

**Affiliations:** 1 Johns Hopkins University Applied Physics Laboratory, Laurel, Maryland, USA

Summary:

 The systematic scoping review analyzed serological studies and assessed their ability to quantify pathogen burden, which the authors defined as a person’s past exposure to infections, and can inform future research on pathogen burden and disease outcomes. The review included 50 pathogens, which were analyzed across 74 studies extracted from three major scientific databases. The study concluded that pathogen burden was not explicitly quantified in most studies, as only binary outcomes (infected or not) were reported. Additionally, there was no strong scientific or clinical rationale for methodological selection.

 Main Comments:

 Overall, the authors attempt to answer an important question about the pathogen burden on the individual and whether understanding past infections could help mitigate the risk of non-communicable diseases caused by pathogens. The study is comprehensive and rigorous in its exploration of serological studies and includes a variety of pathogens. However, I have a few comments that I would like to see addressed before indexing:

- The study selection process appears rigorous, but I would like to see more documentation of the process itself. For example, was there documentation of disagreement among reviewers regarding inclusion, or a PRISMA-ScR checklist associated with this study (See https://www.prisma-statement.org/scoping)?
- Not really clear on what the section “Definitions of pathogen burden” on page 7 is contributing. It seems there should be a table of potential or already-defined definitions.
- Was there an assessment of study methodologies to measure serology? Will test accuracy be an issue for studies trying to understand pathogen burden?
- In the “Rationale behind pathogen scores” section, what was the rationale exactly? I’m not sure I can trace where each study lists its rationale. I would like to see more critical appraisal of the study rationale. Examples would be good in this section.
- The discussion could be strengthened by more clearly outlining how the findings inform clinical or public health practice, especially changes to health surveillance that could improve understanding of pathogen burden.

Minor Comments:

- Is there a justification for including studies with >3 pathogens? Why were studies with two pathogens not included?

Are the rationale for, and objectives of, the Systematic Review clearly stated?

Yes

Is the statistical analysis and its interpretation appropriate?

Yes

If this is a Living Systematic Review, is the ‘living’ method appropriate and is the search schedule clearly defined and justified? (‘Living Systematic Review’ or a variation of this term should be included in the title.)

Not applicable

Are sufficient details of the methods and analysis provided to allow replication by others?

Partly

Are the conclusions drawn adequately supported by the results presented in the review?

Yes

Reviewer Expertise:

Computational Epidemiology, Public Health, and Systems Modeling and Simulation

I confirm that I have read this submission and believe that I have an appropriate level of expertise to confirm that it is of an acceptable scientific standard, however I have significant reservations, as outlined above.

# Response

**Published:** 2026-05-18

**Authors:** Anne Suffel

**Affiliations:** Department of Non-Communicable Disease Epidemiolog, London School of Hygiene and Tropical Medicine Faculty of Epidemiology and Population Health, London, England, UK

**Comment 1:** The study selection process appears rigorous, but I would like to see more documentation of the process itself. For example, was there documentation of disagreement among reviewers regarding inclusion, or a PRISMA-ScR checklist associated with this study (See https://www.prisma-statement.org/scoping)?

**Authors' response:** In addition to the PRISMA flow chart, we have now added a completed PRISMA-ScR checklist in the supplementary information.  

**Comment 2:** Not really clear on what the section “Definitions of pathogen burden” on page 7 is contributing. It seems there should be a table of potential or already-defined definitions. **Authors' response**: The section “other pathogen burden definitions” summarises statistical approaches by various studies which go beyond scores based on pathogen counts or categorisations based on antibody titre.  

 We believe this section is necessary to better describe more complex approaches such as weighted regression indices or latent class analysis which don’t fit into the other categories, i.e., counting the number of seropositive pathogens or counts based on antibody titres. Table 1 summarises all included studies ordered by different approaches taken, e.g., Studies with pathogen index as count, Studies using a count as pathogen burden and titres, other approaches for pathogen burden.  

**Comment 3:** Was there an assessment of study methodologies to measure serology? Will test accuracy be an issue for studies trying to understand pathogen burden?

**Authors' response:** We did not critically assess the methodologies to measure serology as the focus of this study was to identify how pathogen burden has been defined in epidemiological research studies. Scoping reviews do not typically assess risk of bias formally as they aim to map the evidence rather than to provide a definitive answer to a research question (Munn et al. (2018)).  Nevertheless, we agree that test accuracy is an important consideration for future studies and systematic reviews of studies of pathogen burden. We have added the following text to the discussion to highlight this issue: “We did not assess risk of bias in the included studies as the main interest of this review was to assess the scope of methods used to conceptualise pathogen burden rather than to assess the quality of the epidemiological research. *The quality of serological assays evolves over time; and sensitivity and specificity vary by the assays used. Test accuracy should be considered in future epidemiological studies using pathogen burden and when critically appraising the findings of such studies in systematic reviews*.”   Munn Z, Peters MDJ, Stern C, Tufanaru C, McArthur A, Aromataris E. Systematic review or scoping review? Guidance for authors when choosing between a systematic or scoping review approach. BMC Med Res Methodol. 2018 Nov 19;18(1):143. doi: 10.1186/s12874-018-0611-x. PMID: 30453902; PMCID: PMC6245623.  

**Comment 4:** In the “Rationale behind pathogen scores” section, what was the rationale exactly? I’m not sure I can trace where each study lists its rationale. I would like to see more critical appraisal of the study rationale. Examples would be good in this section.

**Authors' response:** We agree with the reviewer that a more detailed description and critical appraisal for the rational of defining pathogen burden would be crucial. However, none of the included studies provided a scientific rationale for the approaches taken and neither was this available in the review paper they referred to. The cited review paper states the following: “ *There is at present no standard definition of infectious burden. There is no consensus as to which organisms or clinical infections should be considered, or whether optimal measures should be based on serological evidence of past exposure, or on a history of past exposures derived from questionnaires, or from independent sources of information, such as chart review or administrative databases.” *From: Elkind MS. Infectious burden: a new risk factor and treatment target for atherosclerosis. Infect Disord Drug Targets. 2010 Apr;10(2):84-90. doi: 10.2174/187152610790963519. PMID: 20166973; PMCID: PMC2891124. Hence, we concluded that referencing the Elkind review from 2010 is not sufficient justification for any of the approaches taken.  

**Comment 5: **The discussion could be strengthened by more clearly outlining how the findings inform clinical or public health practice, especially changes to health surveillance that could improve understanding of pathogen burden.

**Authors' response:** We agree with the reviewer that research on the relationships between pathogen burden and other health outcomes could improve clinical practice and public health. We added the following sentence to the discussion: “Identifying specific patterns of pathogens and infection history in relation to other health outcomes can improve the prevention of both infectious diseases and associated long-term health outcomes. Enhanced surveillance of relevant pathogens, combining repeated serological assays with clinical data, may support risk assessment and guide vaccination and other preventive strategies. “  

**Comment 6:** Is there a justification for including studies with >3 pathogens? Why were studies with two pathogens not included?

**Author's response: **This paper aimed to provide an overview of definitions for pathogen burden arising from the challenge of testing several pathogens at the same time with multiplex assays. We selected ≥3 pathogens pragmatically to reflect more complex definitions. This is stated in the methods section: “We chose studies with at least three pathogens to capture studies with more complex exposure/outcome definitions of multiple infections.”
